# Editorial: Rising stars in cardiac rhythmology: 2023

**DOI:** 10.3389/fcvm.2023.1351604

**Published:** 2024-01-10

**Authors:** Michael Spartalis, Julia W. Erath, Bart A. Mulder, Bert Vandenberk

**Affiliations:** ^1^ 3rd Department of Cardiology, Sotiria Thoracic Diseases General Hospital, National and Kapodistrian University of Athens, Athens, Greece; ^2^ Division of Clinical Electrophysiology, Department of Cardiology, University Hospital Frankfurt, J. W. Goethe University, Frankfurt, Germany; ^3^ Department of Cardiology, University Medical Center Groningen, Groningen, Netherlands; ^4^ Department of Cardiology, University Hospitals Leuven, Leuven, Belgium; ^5^ Department of Cardiovascular Sciences, KU Leuven, Leuven, Belgium

**Keywords:** electrophysiology, catheter ablation, cardiac arrhythmias, cardiac pacing, device therapy

**Editorial on the Research Topic** Rising stars in cardiac rhythmology: 2023

This editorial showcases the compilation of articles published in Frontiers in Cardiovascular Medicine: Rising Stars in Cardiac Rhythmology 2023. Identifying the future leaders in Cardiac Rhythmology is essential for protecting the future driving force of innovation. The objective of this Research Topic is to facilitate the recognition of the excellent contributions made by early-career researchers. The publications in this collection were chosen based on their advancement in the field of electrophysiology and the research potential of the investigators. We highlight the accomplishments of young researchers who excel in the challenging environment of academic research, which involves intense competition for research funding and the need to publish their work consistently (1). These emerging talents exhibit determination, diligence, intellect, and tenacity, the essential attributes required to endure the attrition rate and achieve success. We are delighted to showcase a collection of five original articles submitted by internationally recognized researchers in the early stages of their careers as first authors. The Frontiers in Cardiovascular Medicine: Rising Stars in Cardiac Rhythmology 2023 collection comprises the following publications.

*Use of a new non-contrast-enhanced BOOST cardiac MR sequence before electrical cardioversion or ablation of atrial fibrillation-a pilot study*, by Orbán et al. The left atrial appendage (LAA) thrombus is the primary cause of embolisation in cases with atrial fibrillation (AF) (2). Transesophageal echocardiography (TEE) is the most reliable and widely accepted technique for excluding LAA thrombus (2). This study aimed to assess the effectiveness of a novel non-contrast-enhanced cardiac magnetic resonance (CMR) sequence called BOOST in detecting thrombus in the LAA. It also evaluated the utility of BOOST images in planning radiofrequency catheter ablation (RFCA) compared to contrast-enhanced computed tomography (CT) of the left atrium (LA). The authors also evaluated the patients’ subjective experiences with TEE and CMR. The newly introduced CMR BOOST sequence offers optimal picture quality for ablation planning. The sequence may be useful in excluding larger LAA thrombi, yet its efficacy in identifying smaller ones is restricted. The majority of patients expressed a preference for CMR over TEE in this particular context. This study shows that the CMR BOOST sequence has the potential to become a preferred alternative for TEE in the future. This is due to its high level of accuracy and its consideration of patients’ preferences.

*Associations of adenovirus-reactive immunoglobulins with atrial fibrillation and body mass index*, by Gumanova et al. Adenovirus (AdV) has been implicated in the development of AF (3). The objective of this investigation was to assess the correlation between AdV-specific immunoglobulins G in the serum (AdV-IgG) and AF. The authors demonstrated a distinct and immediate correlation between AdV-IgG-positive reactivity and AF. Furthermore, a sequence of human and animal investigations has demonstrated correlations between specific human AdV types and obesity (4). These viruses have been suggested as a potential catalyst for the obesity pandemic (4). The data from this investigation showed that the prevalence of obese patients was around three times higher in AdV-IgG-positive patients in the group with AF compared to AdV-IgG-negative patients in the same group. These differences were not detected in the control group. The study found a direct correlation between AF and body mass index (BMI), indicating that adenoviral infection may be a possible cause of AF. This publication presents new evidence regarding the molecular mechanisms of infection-induced arrhythmogenesis, indicating that adenoviral infection could be a new etiological factor for AF.

*Remote monitoring of implantable loop recorders reduces time to diagnosis in patients with unexplained syncope: a multicenter propensity score-matched study*, by Russo et al. Insufficient data exists on the remote monitoring (RM) of implantable loop recorders (ILRs) in patients experiencing unexplained syncope and whether this method provides improved diagnostic capabilities (5). This study assessed the impact of remote monitoring (RM) in recipients of ILR who experienced unexplained syncope, aiming to detect clinically significant arrhythmias at an early stage. The results were compared to a historical cohort that did not undergo RM. In the propensity score-matched comparison between patients with unexplained syncope who underwent RM of ILR and a historical cohort, it was found that RM of ILR was linked with a 2.5-fold increased likelihood of assessments for clinically significant arrhythmias compared to biannual in-office follow-up visits. Rapid diagnosis is crucial for various reasons, such as prompt commencement of therapy, ensuring safety, and enhancing the quality of life. As a result, a 2.5-fold higher chance of diagnosis over a median 22-month follow-up found by the authors may have obvious implications for care quality and related costs.

*Feasibility and safety of cavotricuspid isthmus ablation using exclusive intracardiac echocardiography guidance: a proof-of-concept, observational trial*, by Debreceni et al. Catheter ablation is the recommended therapeutic approach for typical atrial flutter (AFL), however it can provide difficulties due to anatomical abnormalities (6). Utilizing 3D electroanatomical mapping systems (EAMS) has resulted in a decrease in fluoroscopy exposure during AFL ablation procedures (6). Intracardiac echocardiography (ICE) has demonstrated advantages in minimizing radiation exposure during AFL ablation (7). Nevertheless, there is insufficient information regarding the practicability of ICE-guided, zero-fluoroscopy AFL ablation in the absence of EAMS use. This research indicates that performing catheter ablation for typical AFL without the use of fluoroscopy and relying solely on ICE for guidance is both possible and safe. Moreover, the findings of this study indicate that this approach has no substantial effect on procedural data, safety, or effectiveness in comparison to typical ICE-guided AFL ablations. It is crucial to minimize or entirely eliminate the use of fluoroscopy because even low doses can be harmful, and there is no clearly defined level of radiation exposure that is absolutely safe. This method can result in a faster and more efficient procedure, completely removing the need for radiation exposure.

*Development and validation of a rapid visual technique for left ventricular hypertrophy detection from the electrocardiogram*, by Somani et al. Methods for detecting left ventricular hypertrophy (LVH) using electrocardiogram (ECG) are difficult to recall and have limited effectiveness (8). This study verified the accuracy of a fast method for detecting LVH and assessed its effectiveness compared to existing methods. The authors provided a new and simplified ECG technique for detecting LVH. This paper evaluated the performance of this technique, along with established LVH strategies, using the biggest dataset that combines ECG and transthoracic echocardiography (ECG-TTE) for this specific purpose. The Witteles–Somani (WS) technique, which utilizes the straightforward evaluation of visibly overlapping QRS complexes in the precordial leads, is somewhat more memorable, quicker to execute, and has comparable efficacy to known techniques. The WS technique is unique because it relies on either high R- and S-voltages between adjacent leads, which favoring high-amplitude isoelectric signals that likely reflect cardiac hypertrophy. It is a highly sensitive, low-cost solution, and an effective screening technique for LVH.

“Rising Stars in Cardiac Rhythmology: 2023” is an outstanding collection of original papers that highlights the contributions of emerging scholars in the field of cardiac electrophysiology. This Research Topic includes original articles by talented young scientists from around the globe showcasing the diverse aspects of electrophysiology. The groundbreaking research presented here has the potential to propel innovations and influence the future of AF management across various domains, spanning from bench to bedside. The publication serves as a forum for these upcoming scientists to disseminate their discoveries and perspectives, thereby enhancing the existing knowledge in the field of electrophysiology and facilitating groundbreaking breakthroughs. This statement recognizes their notable accomplishments and commemorates their capacity to emerge as future pioneers in their respective domains.
